# Population-Based Studies in Dementia and Ageing Research: A Local and National Experience in Cambridgeshire and the UK

**DOI:** 10.1177/15333175221104347

**Published:** 2022-08-24

**Authors:** Carol Brayne, Yu-Tzu Wu

**Affiliations:** 1 School of Clinical Medicine, 2152 University of Cambridge, Cambridge, UK; 2 Population Health Sciences Institute, 5994 Newcastle University, Newcastle upon Tyne, UK

**Keywords:** population-based studies, observational research, epidemiology, ageing, lifecourse

## Abstract

Dementia has been recognised as a key challenge in many ageing societies across the world. Several population-based studies have been developed to investigate dementia and cognitive ageing from perspectives of biology, health, psychology and social sciences. However, there is a need to provide a better understanding of ‘contexts’, the circumstance where these ageing populations existed, and heterogeneity within and across the populations in different time and places. In this article, we summarise some examples of earlier population-based studies undertaken by our research groups in England and Wales and their contribution to the epidemiology of dementia, neuropathology, cognitive and mental health in older age. We also describe how these studies illustrated variation among ageing populations and changes in their health conditions across time and place. These findings highlight the contribution that population-based studies can make, along with the vital to incorporate contexts in ageing research. A lifecourse approach within social context is needed to integrate life experiences, social circumstances, and multiple dimensions of cognition, functioning, physical health and wellbeing over the ageing process. We also discuss how evidence from population-based studies can support various international initiatives on dementia, healthy ageing and Sustainable Development Goals and facilitate tailored approaches for diverse populations across global societies.

## Backgrounds

Dementia has been recognised as one of key challenges related to population ageing.^1,2^ In recent years, there has been a growing interest and investment in dementia research.^3^ A variety of studies have been established to investigate the epidemiology of dementia and ageing across the globe and measures for cognitive function have been widely incorporated into existing cohort studies.^4,5^ The development of computing and data sciences also have facilitated exploitation of large health and biomedical databases such as Clinical Practice Research Datalink (CPRD) in the UK, biobanks from large volunteer cohorts and health insurance data.^6-8^ In addition, data portal and harmonisation have become popular approaches to integrate existing studies of dementia and ageing and allow researchers to identify or create comparable variables and address their research questions.^9,10^ Secondary data analysis is encouraged by such data infrastructures: large sample sizes, numerous variables and complex data with ability to address lack of statistical power. Given the large amount of available data, traditional epidemiological approaches of setting up a new population-based study may seem to be outdated and unnecessary. But the danger of assumptions that routine data and volunteer studies can overcome the biases inherent within these datasets should be carefully considered.^11,12^ The value of population-based studies remains with ability to anchor other approaches to what is happening within populations recruited on the ground.^13,14^

A population-based study is designed to address research questions in a defined population.^15^ The study population is a representative sample of a defined population and therefore its findings can be applied to the whole target population (i.e., external validity). This key feature is essential when estimating unbiased distributions of health conditions and their risk factors and investigating unbiased relationships between variables.^16^ For dementia research, population-based studies are particularly important as dementia is a syndrome of deterioration in cognitive function.^17^ Identification of people with dementia can be affected by the nature of study recruitment, measurement methods for cognitive function, diagnostic criteria, subjective clinician opinions, and characteristics of individuals such as education, health status and medical history.^18^ Therefore studies targeting the whole population, or populations of particular interest, are needed to describe distributions of cognitive function, estimate the prevalence and incidence of dementia in whole populations, and those that have been neglected to date, and investigate risk and protective factors and their population attributable factions. In addition, population-based studies are important to inform the context of diverse ageing populations across time and regions.

Over the last century, many societies in the world have experienced extending life expectancy and growing numbers of older people, in particular the oldest old (age≥85).^19,20^ This may increase diversity within and across ageing populations. Dementia, a key disorder closely associated with older age, is strongly related to other changes that occur increasingly with age such as functional impairment, falling, incontinence, frailty and multimorbidity. The context of historical, societal and cultural factors can influence clinical practices and detection of psychological and behavioural symptoms related to dementia as well as affect individual life experiences, living conditions and health across the lifecourse.^13,21^ There is a need to have a better understanding of older people in diverse settings and examine dementia and cognitive function in the contemporary context. Such information can only be provided from population-based studies, which collect primary data from a representative sample of a defined population in specific time and place.

This review first describes some examples of population-based studies undertaken by our research groups in England and Wales. We illustrate a journey from a regional study in East Cambridgeshire to multicentre longitudinal cohort studies in the UK and summarise their key findings. The discussion focuses on lessons learnt from these existing studies and highlights the value of population-based studies. We argue population-based studies are crucial to provide a comprehensive picture in diverse older populations and inform the context of dementia and ageing research and relevant policy planning.

## Examples of Population-Based Studies in Cambridgeshire and UK

### The East Cambridgeshire Study

In the 1980s, a small group of population-based studies started to investigate dementia and cognitive decline in western Europe and address limitations of the earlier prevalence estimates derived from medical records.^22^ This epidemiological investigation was one of the pioneering studies.^23^ The study focused on 365 women aged 70-79 in rural East Cambridgeshire^24^ including one town and four villages with a relatively stable population. This study focused on women with all those aged 70-79 in this area being approached over a period of 8 months from 1985-6. One hundred and eighty-five participants in the age group 70-74 and 180 in the 75-79 were interviewed (response rate 91%). A structured psychiatry interview was developed mainly based on the Cambridge Examination of Mental Disorders in the Elderly (CAMDEX)^25^ comprising measures for cognitive function and neuropsychological battery (Mini-Mental State Examination (MMSE)^26^ and Cambridge Cognitive Examination (CAMCOG)^27^), observation of mental state, physical examination and blood samples. The interview was carried out in participants’ residences. For the first time in such a study, informant interview was also included on almost all participants, interviewed separately and explicitly not a spouse to avoid bias. Clinical diagnosis of dementia was made according to the CAMDEX guidelines. A random subset was interviewed by a consultant psychiatrist trained in the administration of the Geriatric Mental State (GMS), with application of the Automated Geriatric Examination Computer Assisted Taxonomy (AGECAT) dementia algorithm.^28^ The follow-up study did provide the opportunity to study outcomes, with publication of some of the approaches that are still used today such as simple and choice reaction time, National Adult Reading Test (NART) IQ (used for first time in community in this study) and dementia at 5 years.^29-31^

From a contemporary point of view, this study may seem to be small and limited to a regional subgroup. However, this population-based study addressed some important issues in dementia research. Instead of relying on medical records or patient data in clinical settings, this study collected primary data from community-based older people and highlighted continuum of cognitive function and complexity of dementia syndrome among the ‘whole’ population.^13,30^ The structured psychiatric interviews indicated the potential for standardised approach to be employed in large epidemiological investigations and included what is now seen as deep phenotyping in that there was a neurological and vascular examination, as well as a blood draw.

### Cambridge City Over-75s Cohort Study (CC75C)

CC75C (www.cc75c.group.cam.ac.uk) is a longitudinal cohort study of people aged 75 or above in Cambridge, UK.^32^ Established in 1985 alongside the East Cambridgeshire study described in 2.1, the study included both people living in community and care settings and focused on investigating the epidemiology of dementia and longitudinal changes in cognitive function, health and wellbeing among older people. Eligible participants were identified through primary care registrations with a selection of geographically and socially representative general practices in the area.

The baseline interview included 2609 participants with a 95% response rate. Ten follow-up waves were carried out over 28 years. For each wave, CAMCOG (covering the MMSE items), was used to assess cognitive function of the participants. A two-stage approach was employed to identify dementia cases using MMSE (screening) and detailed psychiatric interviews including CAMDEX, additional neuropsychological assessments and tests (clinical diagnosis).^33,34^ In addition to measures for cognitive function, a wide range of longitudinal data were collected including sociodemographic variables, activities of daily living, use of health and social services, health status and medication, subjective well-being and quality of life near the end of life (involving relatives or carers of the participants). The study also collected biological resources, including blood and saliva samples, bone fragility (the sixth wave) as well as over 240 brain donations since 1988.

Compared to the East Cambridgeshire study, CC75C expanded from dementia and cognitive function to a variety of topics in ageing research covering biology, medical and social sciences. The longitudinal data over nearly three decades provided rich information on identifying changes within individuals over time. The response rates of follow-up waves remained high in those who were alive. The representative sample of older people and their relatives has created an extensive resource of quantitative and qualitative data on dementia, depression, cognition, function, delirium and healthy ageing. This study was ground-breaking with regard to pioneering population-based brain collection after participants have died. This was received very positively by those who took part, and their families resulting in successful donation of around 250 brains for biomedical research after our respondents’ deaths (www.cfas.ac.uk/participant-info/mind-over-matter/).

### Cognitive Function and Ageing Studies (CFAS)

The Cognitive Function and Ageing Studies are three multicentre population-based cohort studies of people aged 65 or above living in community and institution settings across the UK. Details of CFAS have been described in the study website (www.cfas.ac.uk) and previous publications.^35-38^ The original cohort, Medical Research Council (MRC) CFAS, was established in 1989 and included six centres in England (Cambridgeshire, Newcastle upon Tyne, Nottingham, Oxford and Liverpool) and Wales (Gwynedd). The baseline interview included 13004 participants between 1990 and 1993 and biennial follow-up waves were carried out over the following two decades until 2008. The baseline study population was sampled from primary care registrations, which provide a comprehensive list of eligible participants in the study areas. An introductory letter was sent by local General Practitioners, followed by a visit from a study interviewer. For each centre, 2500 participants were recruited with equal sample sizes of those aged 65-74 years and ≥75 years. Informed written consent was obtained from those who agreed to participate the study. Trained interviewers visited participants’ residences and delivered standardised computerised questionnaires, which included assessment tools for cognitive function and psychiatric symptoms (MMSE, GMS) to allow differential diagnoses of dementia, depressive and anxiety disorders using an algorithmic programme (AGECAT). A two-stage approach of screening and assessment was employed in the prevalence and 2-year incidence estimation. Additional information on socio-demographics, lifestyle, health status and medication was collected in the interviews. Similar to CC75C, MRC CFAS also had a brain donation programme (N≥550) and both are a part of the EClipSE network.^39^

In 2008, a new cohort study, CFAS II, was designed to recruit a representative sample of older population at that time and investigate changes in the epidemiology of dementia and ageing over 20 years. To provide comparable data with the original cohort, three of the MRC CFAS centres (i.e., ‘CFAS I’: Cambridgeshire, Newcastle upon Tyne and Nottingham) were selected in CFAS II. The same recruitment and sampling methods were employed to these areas. The baseline interview included 7796 participants (approximate 2500 per centre) between 2008 and 2011 and a 2-year follow-up was carried out between 2011 and 2013. The only change in CFAS II was the one-stage approach of dementia diagnosis. While the same standardised tools were used to measure cognitive function and psychiatry symptoms, the combined screen and assessment interview, which was developed in later follow-up waves of MRC CFAS, allowed to avoid loss to follow up between screening and assessment stages and provide the differential diagnoses for all the CFAS II participants and straightforward prevalence and incidence estimates. The same study design was applied to CFAS Wales, a population-based cohort of people aged 65 or above in rural (Gwynedd and Ynys Môn) and urban (Neath Port Talbot) areas of Wales (cfaswales.bangor.ac.uk). The baseline interview of CFAS Wales was carried out between 2011 and 2013 and included 3593 participants. In addition to the standard CFAS II interview, the Welsh cohort collected more detailed information on social activity and participation, adaption and resilience, environmental factors and bilingualism (Welsh/English).

Built on experiences on the East Cambridgeshire study and CC75C, CFAS was expanded to multicentre population-based studies focusing on the epidemiology of dementia and cognitive function in the UK older population. CFAS included independent study populations geographically representative at two time points and used the same study designs and research methods to measure cognitive function and dementia. It also used the methods developed in CC75C to establish a brain donation programme that is still in place. The findings had the potential to be mapped to the nation, using geographical and demographic approaches. This enables investigations of potential changes in prevalence, incidence, risk and protective factors over time.

## Key Findings

### Prevalence and Incidence

Population-based studies are essential to provide information on the descriptive epidemiology of dementia. All three population-based studies were designed to estimate the prevalence and incidence of dementia in defined populations at specified time points. The two studies established in the mid-1980s focused on the older old (age≥70). The East Cambridgeshire study was based in rural settings and CC75C was in urban areas of Cambridge. The two studies found that the prevalence of dementia increased with older age.^33,40^ In the East Cambridgeshire study, the prevalence of women was 4.3% in age 70-74 and 11.7% in age 75-79. In CC75C, the prevalence was estimated to be 10.5% for people aged 70 or above.

The aim of CFAS was to provide nationwide estimates for dementia prevalence and incidence in people aged 65 or above at two time points (1991 and 2011). Based on MRC CFAS, the prevalence standardised to the 1991 population was 6.6%. The prevalence standardised to the 2011 population was 6.5% based on CFAS II. In addition to estimates for the overall population, the results also inform the potential variation within the population, including age- and sex-specific estimates as well as those living in community and care settings. Since the similar study designs and measurement methods were used in MRC CFAS and CFAS II, the estimates from the three geographical areas in England were comparable and suggested decrease in the prevalence and incidence over time.^35,36^ The prevalence (standardised to the 2011 population) decreased from 8.3% to 6.5%. The incidence (standardised to the 2011 population) was 20.0 per 1000 person years in the earlier cohort and 17.7 in the more recent cohort. The CFAS data are also involved in the recent Alzheimer Cohort Consortium (ACC), which harmonised data on dementia incidence across ageing cohorts and suggested a decreasing incidence rate in Europe and North America.^41^

### Risk and Protective Factors

Identifying risk and protective factors is a key focus of epidemiological research. Although it is often assumed that investigation of the relationships between exposure and disease does not require samples to be representative,^42^ highly selected samples can still lead to substantially biased estimates of causal associations.^12,14^ In addition, only population-based studies can estimate the prevalence of risk and protective factors and examine variation within target populations.

The earlier two studies in the Cambridge areas identified older age as a key risk factor for dementia.^24,34^ In addition to demographic factors, CC75C suggested several vascular risk factors for incident dementia (history of heart attack, transient ischaemic attacks and cerebrovascular accidents) but found no associations with lifestyle factors (smoking, alcohol consumption, cognitive and physical activity).^43,44^

CFAS collected a wide range of potential factors related to dementia. The earlier results from MRC CFAS suggested several risk factors in sociodemographic (older age, women, low education attainment) and health domains (poor self-rated health, stroke and Parkinson’s disease). Lifestyle factors including alcohol consumption and smoking were not associated with incidence of dementia.^45^ The CFAS II analysis identified similar risk and protective factors in sociodemographic (age, sex, education) and health domains (poor self-rated health, stroke and Parkinson’s disease) as well as some different factors including deprivation, unskilled occupation, widowhood, living in care settings, functional impairment and loneliness.^46^ Based on more detailed information on lifestyle factors in CFAS II, abstaining from alcohol, current smoking and physical inactivity were found to be associated with increased risk of incident dementia. The strength of associations for risk and protective factors generally remained similar but the prevalence of risk and protective factors varied across the two cohorts at different time points.^46^

Using enriched data in MRC CFAS, a cognitive lifestyle score was developed to incorporate education (early life), occupational complexity (midlife) and social engagement (later life) throughout different stage of life.^47^ A high cognitive lifestyle score was associated with a lower risk of incident dementia and the combination of education in young age and any of the two components in mid or later life were also protective of dementia.^47^

### Neuropathology and Neurobiology

Risk and protective factors for dementia have been identified in these population-based studies in the UK and other ageing cohorts across the world. However, few studies were able to investigate underlying mechanisms between these factors and dementia. The brain donation programmes in CC75C and CFAS and their harmonised dataset with Vantaa 85+, a population-based study of people aged 85 or above in Finland, have formed the EClipSE collaboration to provide a unique opportunity to address this gap and allowed investigation of brain neuropathology and neurobiology in the general population.^39,48^

Based on the brain donation data in MRC CFAS (age 69-103), the pathological features of Alzheimer’s disease and dementia were identified in both people with and without dementia. The associations between the Alzheimer’s type pathology and dementia reduced with older age.^49^ The results highlight complexity of brain ageing in late onset dementia and the need of shift away from single features and investigate broader pathological markers. The EClipSE project examined a wide range of pathologies such as Pick bodies, severe neuronal loss, gliosis, and granulovacuolar degeneration in the cortex and/or hippocampus and found that most of them had independent associations with dementia in addition to classical markers of plaques and tangles.^50^ The findings have also contributed to the debate about a new ‘diagnosis’ limbic-predominant age-related TDP-43 encephalopathy (LATE), based on findings in the hippocampus – the TDP-43 protein associated with hippocampal sclerosis.^51^ The EClipSE data were also used to examine whether education and active cognitive lifestyle were related to dementia and brain pathologies. Although high education attainment was found to be a protective factor for dementia, it was not associated with neurodegenerative or vascular pathologies but only brain weight.^52^ Similarly, active cognitive lifestyle was not related to Alzheimer’s type pathology but was associated with greater neuronal density in prefrontal lobe.^53^ The results support the hypothesis of cognitive reserve that education and active cognitive lifestyle facilitate individuals to compensate for burden of neurodegenerative and vascular pathologies.

### Beyond Dementia: Continuum of Cognitive Function and Transitions, Mild Cognitive Impairment and Delirium

The complexity of dementia diagnosis has been widely recognised in research and clinical practice. Variation in diagnostic criteria and implementation of cognitive and neuropsychological tests can influence identification of dementia cases.^54,55^ Instead of using a binary definition, population-based studies have provided a comprehensive picture informing continuum of cognitive function in the whole population and longitudinal trajectories of cognitive function.

All the three population-based studies provide evidence on distributions, trajectories and changes of cognitive function in older age. The East Cambridgeshire study and CC75C both reported the distributions of MMSE scores in the target population.^40,56^ The results provided population norms of this specific cognitive test and indicated variation within the population by sociodemographic characteristics. In CC75C and MRC CFAS, longitudinal data on cognitive function were modelled to indicate individual trajectories over time and examine potential variation across subgroups.^57,58^ In addition to trajectories, data from MRC CFAS were also used to investigate transition of cognitive states, focusing on how individuals transited between different cognitively impaired states over time.^59,60^ This approach was used to investigate the impact of cognitive lifestyle on transition states and the results showed that an active cognitive lifestyle was associated with reduced risk of developing cognitive impairment and increased likelihood of recovering from slightly impaired back to non-impaired state.

Mild cognitive impairment (MCI) has been considered as a early stage of dementia but the definitions of this condition vary across the literature. Using the MRC CFAS data, eighteen definitions of MCI were mapped to the study population.^61^ The prevalence of MCI was found to vary considerably (2.5-41.0%) across different definitions and their associations with progression to dementia were inconsistent.^62,63^ The comparison of the CFAS I and II data also reported variation in the MCI prevalence across different definitions and most definitions showed decreasing and stable prevalence over the two decades.^64^

Delirium, a neuropsychiatric syndrome of altered consciousness and attention with cognitive emotional and behavioural symptoms, is frequent in older people with medical or surgical conditions.^65^ Using the data from EClipSE collaboration, delirium was found to be a strong risk factor for dementia and cognitive decline. While classic neuropathology related to dementia was not mediated the association, combination of delirium and dementia pathology was associated with greater decline in cognitive function than those with single condition.^66,67^

## Ageing in Context

From the Cambridge areas to the multicentre nationwide study, the three population-based studies were designed to provide a comprehensive picture of dementia and cognitive ageing in older people. The whole population approach showed full spectrum of cognitive function and brain health in ageing populations. Given the diversity within and between older populations and ageing processes, the findings highlighted the complexity of measuring dementia, MCI and cognitive decline and identifying risk factors and pathological markers related to dementia. Compared to volunteer cohorts and health administrative databases, these studies emphasised robust sampling framework, quality control of measurement methods and in-depth analyses of uncertainty (non-response rate, loss to follow-up, missing data). The strengths of these population-based studies have provided robust evidence on the context of ageing population across time and places in the UK.

The comparison of CFAS data indicates changing profiles of cognitive function and health in older populations over two decades. While the prevalence and incidence of dementia decreased across the two cohorts, socioeconomic inequalities within the older populations were found to increase over time.^68,69^ To provide a better understanding of these changes, it is necessary to adopt a lifecourse approach and investigate how individual life experiences, living conditions, cultural, economic and societal factors can influence cognitive health in later life. Individual level factors such as education, lifestyle and health conditions have been recognised as important risk and protective factors but their roles in individuals’ life may change over time, generations and contexts. In particular, the results from these studies support the concept of cognitive reserve, which focuses on individual differences in resilience to brain pathologies.^70^ The risk and protective factors at different life stages may hinder or facilitate development of compensatory approaches against brain damage but their effects might differ across contexts.^52^

Population-based can also provide opportunities to investigate factors beyond individual level and distal determinants that modify known risk and protective factors (such as physical activity). For example, geolocation information in CFAS were used to integrate with data on the built environment and investigate the potential impact of environment on cognitive health in older people.^71,72^ Several recent studies have also modelled exposure to air pollution based on residential locations and suggested their associations with increased risk of dementia.^73^ This approach can add values to existing cohort studies and provide additional information on spatial and environmental contexts of ageing populations.

## Conclusions

Over the last decade, dementia has been a key topic in research, politics, media and the public. Several international initiatives and national plans have been established to raise awareness and political commitment to address this challenging condition.^74,75^ To support evidence-based policy planning, novel population-based studies are still needed now and in the future. With well-defined target populations and roust study designs, population-based studies can provide valuable evidence that informs the epidemiology of dementia in the fields of public health, clinical medicine, neuropathology and biology and highlight heterogeneity within the ageing population and changing profiles of cognitive health across time and places. The whole population approach is essential to provide up to date and contextual information on dementia and ageing populations and facilitate tailored approaches for diverse populations within and across countries, regions and societies.^76^ The unique value of population-based studies cannot be replaced by large volunteer cohorts and health administrative databases. Although big data analytics can facilitate utilising opportunistic data and testing complicated relationships between a large number of variables, the underlying ‘out of context’ assumption and non-representative samples need to be considered in interpretation and contextualisation of the findings. On the other hand, the findings from population-based studies can provide straightforward messages that inform relevant health and social policies in specific populations, time and places.

Evidence from population-based studies shows the interrelationships between physical, mental and cognitive health in older populations. It is important to integrate dementia and other aspects of ageing research such as stroke, disability and frailty as well as consider dementia in a wider context of healthy and sustainable ageing. The recent healthy ageing framework has started to shift away from ‘what diseases and health conditions in an older person’ (i.e., intrinsic capacity) and focus on ‘what a person can do in older age’ (i.e., functional ability) and ‘how to support a person to maintain functional ability and wellbeing’ (i.e., environment).^77,78^ Chronic conditions, including dementia, can have substantial impact on all aspects of functional ability (abilities to meet basic needs; to learn, grow and make decisions; to build and maintain relationships; to be mobile; and to contribute to society).^78^ Yet a supportive environment may facilitate people living with physical and mental health conditions to maintain these abilities and reduce risk of cognitive decline, dementia, disability and poor quality of life. The latest Sustainable Development Goals (SDGs) outline a shared blueprint for peace and prosperity for people and the planet.^79^ All the SDGs indicators are relevant to population health and can have substantial impacts on ageing populations.^80^ Older people are particularly vulnerable to the drivers of poor health such as poverty, gender inequality, pollution and climate changes and their prolonging influence can affect life and health of the future generations of older populations.^81,82^ Achieving the vision of SDGs will promote health and wellbeing for all at all age and facilitate addressing challenges of dementia and healthy ageing.
